# Hope level and associated factors among older people living with HIV/AIDS: a cross-sectional study

**DOI:** 10.3389/fpubh.2024.1371675

**Published:** 2024-04-17

**Authors:** Chunlan Yu, Yan Wu, Yuli Zhang, Mei Li, Xin Xie, Longsheng Xie

**Affiliations:** ^1^Outpatient Department, The Affiliated Hospital of Southwest Medical University, Luzhou, China; ^2^Department of Hepatobiliary Surgery, The First Branch of The First Affiliated Hospital of Chongqing Medical University, Chongqing, China; ^3^Department of Gynecology, The First Branch of The First Affiliated Hospital of Chongqing Medical University, Chongqing, China; ^4^Department of Nephrology, The Affiliated Hospital of Southwest Medical University, Luzhou, China

**Keywords:** older people, HIV, hope, stigma, social support

## Abstract

**Background:**

In China, little is known about the hope level of older people living with HIV/AIDS (PLWHA).^1^ This study was to examine the hope level of older PLWHA in China and identify related factors.

**Methods:**

This cross-sectional study was conducted in Sichuan province in China among older PLWHA.^2^ A standardized self-report questionnaire, the Herth Hope Index, was adopted. Multiple linear regression was used to identify factors influencing hope level. *p*-values <0.05 were considered statistically significant.

**Results:**

There were 314 participants with an average age of 64.5 (SD ± 8.7). Most of the participants were males (72.6%), primary school and below (65.9%), rural household registration (58.6%) and married (64.3%). More than half of the older adults had pension insurance, had a monthly income of more than RMB 1,000 and considered themselves to be in good health. About 80% confirmed being diagnosed for more than a year and disclosed their HIV status to family and friends. The majority of the population had low medium social support (79%). More than 80% had moderate and severe HIV stigma. Many older PLWHA had medium and high levels of hope, with an average score of 34.31 (SD ± 4.85). Multiple linear regression showed that having pension insurance (*β* = 1.337, *p* = 0.015), longer diagnosis (*β* = 0.497, *p* = 0.031), better self-reported health (*β* = 1.416, *p*<0.001) and higher levels of social support (*β* = 2.222, *p* < 0.001) were positively associated with higher levels of hope. HIV stigma (*β* = −1.265, *p* < 0.001) was negatively correlated with hope level.

**Conclusion:**

The hope level of older PLWHA is good, but there is still room for improvement, and its hope is related to multiple factors. Therefore, the AIDS-related healthcare sector should pay special attention to the hope of older PLWHA, help them to improve their health, provide financial assistance and social aid to those with financial difficulties, and take measures to reduce HIV stigma, improve family support for the older adults, and guide the older adults to adopt a positive approach to life.

## Introduction

There is an increasing trend toward an aging population of people living with HIV/AIDS (PLWHA). Highly active antiretroviral therapy (HAART)^3^ has extended the life expectancy of people living with HIV, making AIDS a chronic disease (1). Additionally, HIV infections are soaring among people over the age of 50. According to China CDC Weekly, the proportion of newly HIV-positive males aged 60 and above increased to 18.21% in 2020 (2). There is a growing body of research focusing on issues related to older people living with HIV (PLWHA).

As the UNAIDS 90–90-90 goal for people living with HIV is increasingly being realized in many contexts, the fourth 90, which is to give 90% of people living with AIDS a normal quality of life, has gained increased attention (3). This makes improving the quality of life of people living with AIDS a hot and difficult issue facing public health. One of the challenges in achieving the 4th 90 goal is the mental health of this group. Mental health issues have become the biggest challenge to improving the quality of life of people living with HIV.

AIDS is an incurable, transmissible and stigmatized disease which has a huge physical and emotional impact on those who suffer from it. Mental health problems are common among older PLWHA. Research (4) indicates that older PLWHA are more likely to experience HIV-related stigma and co-morbid mental illness than their younger counterparts. Past studies (5–7) have observed that factors such as HIV stigma, sexual stigma, and age discrimination cause many older PLWHA to experience mood disorders such as anxiety and depression, and that social discrimination and family isolation cause them to lose hope for survival, develop suicidal thoughts, and commit suicidal behavior.

Hope is recognized as an important determinant of mental health recovery (8), which motivates patients to seek treatment. Although hope does not promote healing, it provides the courage to continue to fight for improvement (9). For hopeful patients, the process of facing the disease may be more enjoyable. Hope is a multidimensional and dynamic life force that enables people to look forward to and achieve their individual goals with a positive and optimistic attitude (10). Hope is a protective factor that is thought to be associated with better psychological mental and physical health, better quality of life and well-being, and greater life and happiness (9).

Given the aging trend in PLWHA and the importance of hope, there is a need to understand the level of hope among older people living with HIV which could help healthcare workers take targeted measures to improve their level of hope to promote physical and mental health. To date, almost no studies in China have measured the hope of HIV-infected older people. Thus, the implications of investigating the level of hope among older PLWHA were profound. This study aimed to assess the hope level and related factors for older people in China.

## Methods

### Study design

This cross-sectional study was conducted in Luzhou City, Sichuan Province from May to October 2021, using the Chinese version Herth Hope Index (HHI), Social Support Revalued Scale (SSRS), simplified Berger HIV Stigma Scale (SBHSS) and questions about the demographic information. The study data were collected in the HIV treatment and management departments of health services. Please refer to the Supplementary material for specifics of the questionnaire.

### Participants

Convenience sampling was used in this study because of the state’s protection of PLWHA privacy and the difficulty of the general population to have direct access to this group. Due to the necessity of the study, we obtained permission from the regional CDC to sign a privacy and confidentiality agreement and conducted the questionnaire collection in the field with the assistance of the community HIV follow-up manager. We recruited eligible participants on-site, and the researcher provided instructions for completing the questionnaire. After completing the questionnaire, participants were given some household items as a gift for participation. Inclusion criteria for the research subjects: HIV-positive, aged 50 years or older, having clear consciousness and ability to communicate and understand questions, and being willing to participate in research. People with a mental disorder or poor mental health status were excluded.

### Ethical approval

The study was approved by the research institution’s Human Research Ethics Committee. The researchers signed an AIDS confidentiality agreement. The researcher explained the purpose of the study and its procedures to the participants before the survey. Participants participated in the study voluntarily and could withdraw from the study at any time.

### Measures

Chinese version Herth Hope Index (HHI), Social Support Revalued Scale (SSRS) and simplified Berger HIV Stigma Scale (SBHSS) were used. HHI is currently the most widely used scale. It was translated and introduced by Chinese scholar Professor Zhao Haiping in 1999 and has been widely used in research on hope levels in China (11). The scale consists of 3 dimensions and 12 items, which include temporality and future (T), positive readiness and expectancy (P), and inter-connectedness (I). Each question had four Likert-type options: completely disagree, disagree, agree and completely agree, scoring 1–4 respectively, with a total score of 12–48. Questions 3 and 6 had inverted scores. A higher score means a higher level of hope. Levels of hope were classified according to hope scores. A total score of 12–23 is defined as a low level of hope, 24–35 as a medium level of hope and 36–48 as a high level of hope. SSRS was developed by Chinese scholar Xiao (12). The scale has 10 items with a total score of 12–66, which can be divided into three levels of social support: low (≤22), medium (23–33) and high (>45). SBHSS was derived from the Berger HIV Stigma Scale and scholars from Peking University in China simplified the scale (13). The scale has 15 items with a total score of 15. A score of 0 indicated no HIV stigma, 1–5 indicated mild HIV stigma, 6–10 indicated moderate HIV stigma, and 11–15 indicated severe HIV stigma.

### Statistical analysis

The data were analyzed using SPSS version 21. All variables involved in the study and measurement results were analyzed by descriptive statistics according to the type of variables. The continuous variables were subject to normal distribution using Mean and Standard deviation (SD). Categorical variables were statistically described by frequency (percentage). The dependent variable in this study was the total score of hope which was a normal continuous variable. First, the univariate Pearson correlation test was used to screen statistically significant variables. Then the variables that were statistically significant in the correlation analysis were included in the multivariate linear regression analysis. All analyses were two-sided, and *p*-values <0.05 were considered statistically significant.

## Results

A total of 338 questionnaires were distributed in this study, of which 314 were valid, with a valid response rate of 92.9%. Table 1 shows the characteristics of the participants and univariate correlation analysis of hope level. There were 314 participants with an average age of 64.5 years (SD ± 8.7). Most of the participants were males (72.6%), primary school and below (65.9%) and rural household registration (58.6%). 64.3% of participants were married. Older people living alone had a high proportion (32.8%). Most old people had a monthly income of more than 1,000 yuan and more than half of them had pension insurance. About 80% had been diagnosed for more than 1 year. More than half of the older adults rated their health as good and only 28.7% were in the stage AIDS. Nearly 80% had disclosed their HIV status to family and friends. The average score of social support was 28.8 (SD ± 6.9). The majority of the population had low and medium social support. More than 80% had moderate and severe HIV stigma. A majority of participants had medium and a high level of hope. The average score of hope level was 34.31 (SD ± 4.85).

**Table 1:** Characteristics of the participants and univariate correlation analysis of hope level (*n* = 314).

Variables	Frequency, n (%)	Pearson’s correlation (r)	*p*-value
Gender		−0.115	0.041
Male	228 (72.6)		
Female	86 (27.4)		
Age (years)		−0.012	0.832
50–59	105 (33.4)		
60–69	113 (36.0)		
70–79	84 (26.8)		
≥80	12 (3.8)		
Education level		0.185	0.001
Primary school and below	207 (65.9)		
Junior high school	76 (24.2)		
Senior high school	24 (7.6)		
Junior college and above	7 (2.2)		
Household registration		−0.155	0.006
Urban	130 (41.4)		
Rural	184 (58.6)		
Marital status		−0.064	0.259
Married	202 (64.3)		
Divorced or widowed	99 (31.5)		
Unmarried	13 (4.1)		
Whether is living alone		−0.034	0.544
Yes	103 (32.8)		
No	211 (67.2)		
Monthly income (yuan)		0.241	<0.001
<1,000	116 (36.9)		
1,001–2000	89 (28.3)		
2001–3,000	44 (14.0)		
3,001–4,000	40 (12.7)		
>4,000	25 (8.0)		
Pension insurance		0.235	<0.001
Not have	117 (37.3)		
Have	197 (62.7)		
Diagnosis (years)		0.120	0.034
<1	61 (19.4)		
1–3	140 (44.6)		
3–5	43 (13.7)		
>5	70 (22.3)		
Self-rated health		0.410	<0.001
Very poor	11 (3.5)		
Poor	46 (14.6)		
Fair	85 (27.1)		
Good	109 (34.7)		
Very good	63 (20.1)		
Whether is in the stage of AIDS?		0.047	0.404
No	224 (71.3)		
Yes	90 (28.7)		
Have you disclosed your HIV status to family and friends?		0.000	0.998
No	69 (22.0)		
Yes	245 (78.0)		
Social support level		0.237	<0.001
Low (score ≤ 22)	63 (20.0)		
Medium (score 23–44)	248 (79.0)		
High (score > 45)	3 (1.0)		
Degree of HIV stigma		−0.269	<0.001
No (score = 0)	1 (0.3)		
Mild (score 1–5)	42 (13.4)		
Moderate (score 6–10)	119 (37.9)		
Severe (score 11–15)	152 (48.4)		
Level of HIV hope		-	-
Low (score 12–23)	6 (1.9)		
Medium (score 24–35)	177 (56.4)		
High (score 36–48)	131 (41.7)		

From Table 1, univariate correlation analysis showed that education level (*r* = 0.185, *p* = 0.001), monthly income (*r* = 0.241, *p* < 0.001), having pension insurance (*r* = 0.235, *p* < 0.001), the time diagnosis (*r* = 0.120, *p* = 0.034), self-reported health (*r* = 0.410, *p*<0.001), and level of social support (*r* = 0.237, *p*<0.001) were positively associated with higher levels of hope. Gender (*r* = −0.115, *p* = 0.041), household registration (*r* = −0.155, *p* = 0.006) and degree of HIV stigma (*r* = −0.269, *p* = 0.015) were negatively correlated with hope level.

### Multivariate analysis: factors influencing hope level of older PLWHA

Multiple linear regression identified factors associated with hope level in older PLWHA (Table 2). Having pension insurance (*β* = 1.337, *p* = 0.015), longer diagnosis (*β* = 0.497, *p* = 0.031), better self-reported health (*β* = 1.416, *p* < 0.001), and higher levels of social support (*β* = 2.222, *p* < 0.001) were positively associated with higher levels of hope. HIV stigma (*β* = −1.265, *p* < 0.001) was negatively correlated with hope level. The D-W test value is 1.950.

**Table 2:** Multivariate analysis of hope level in patients with older PLWHA (*n* = 314).

Variables	β	SE	β’	t	*p*-value
(Constant)	25.188	2.202	−	11.437	<0.001
Gender	−0.356	0.533	−0.033	−0.668	0.505
Education level	0.180	0.367	0.027	0.489	0.625
Household registration	0.328	0.565	0.033	0.581	0.562
Monthly income (yuan)	0.311	0.225	0.083	1.387	0.167
Endowment insurance	1.337	0.547	0.133	2.444	0.015
Diagnosis (years)	0.497	0.229	0.106	2.167	0.031
Self-rated health	1.416	0.236	0.314	5.996	<0.001
Level of social support	2.222	0.566	0.191	3.926	<0.001
Degree of HIV stigma	−1.265	0.337	−0.187	−3.757	<0.001

F = 10.372, *p* < 0.001; VIF: 1.029–1.314; R^2^ = 0.235; D-W test value is 1.950.

## Discussion

The study was special which was conducted during the COVID-19 pandemic, which may have had an impact on participants’ psychological states. During the COVID-19 pandemic, factors such as substance use, antiretroviral adherence, social support, financial hardship and economic vulnerability were associated with increased psychological distress of HIV-positive people (14). A study (15) noted that in the COVID-19 epidemic, the occurrence of three negative emotions, inner restlessness, forgetfulness, and exhaustion, were more severe in older AIDS patients than in young and middle-aged patients. Although the COVID-19 epidemic had a negative impact on the psychological status of people living with HIV, the level of hope for older PLWHA in this study remained good.

Our findings suggested that the majority of older PLWHA had moderate or high levels of hope. This is consistent with the results of Chinese studies on the level of hope for PLWHA (16, 17). This result may be due to these facts. First, our study site was in Luzhou, not the center of the COVID-19 outbreak, and the outbreak was not severe. As a result, the local government did not strictly control the city, and ART follow-up treatment of older PLWHA was largely unaffected. Second, ART makes AIDS a chronic disease and reduces the incidence of mortality and related complications. In China, AIDS care policy enables PLWHA to receive free antiviral treatment. In this study, 71.3% of older PLWHA did not progress to the stage of AIDS due to long-term ART. Third, the anti-discrimination publicity of AIDS in various countries around the world has made people have a certain understanding of AIDS, reduced social discrimination of the public against AIDS patients, and thus relieved the psychological pressure on patients. In addition, with the advocacy of the concept of humanistic care, society and AIDS-related healthcare workers (18) attach more importance to the psychological care of PLWHA.

In this study, pension insurance, time of diagnosis, self-reported health status, and level of social support were independent influencing factors for the level of hope. Pension insurance is an important living guarantee for older PLWHA, and those who have it have a stable income every month. As AIDS patients live longer, the potential for other complications and risk factors arises, leading to costly medical care. Studies (19, 20) have shown that complications of AIDS can lead to impaired immune function, which in turn can increase hospitalization rates, hospital expenses, and patients’ financial burden. Pension insurance is a crucial financial security. Many older PLWHA no longer have a source of income from work because of their age, and pension insurance may be their main source of income. Therefore, it is necessary to pay attention to the pension insurance situation of older adults PLWHA, and for those who have no pension insurance and are in poor economic conditions, the government and society could provide economic assistance and social aid, to help the patients raise their level of hope and to promote the patients’ mental health, which in turn will improve their ability to cope with the disease.

In this study, the duration of HIV diagnosis was a protective factor affecting the level of hope. This is contrary to the findings of another study (21) that the longer the disease, the lower the level of hope. Firstly, this may be because, as the disease progresses, older PLWHA have a reduced fear of AIDS, revisit the issue of life and death, and value life more, hence their level of hope is higher. Secondly, compared to other diseases, the country has invested a lot of resources in AIDS prevention and control, and infected people can receive free AIDS treatment drugs. Therefore, even if the duration of the disease is prolonged, patients have basic guarantees for medical treatment and thus have a higher level of hope. This suggests that we can use peer support to conduct a hope intervention for older PLWHA with low hope (22). Let the older PLWHA with long diagnosis and high hope participate in peer assistance and encouragement of the older PLWHA with low hope.

The better the self-reported health status of the older PLWHA, the higher their level of hope. Scioli et al. (23) demonstrated that the total hope scores and hope sub-scores were significantly correlated with various dimensions of self-reported health status, which is consistent with our findings. This may be because the better the self-rated health of the older adults, the less burden of AIDS-related symptoms they are likely to bear and the less impact the disease has on the older adult person’s life. Hope is an emotion that may exert powerful effects on health (23, 24). Health promotes hope and hope influences health, thus achieving a virtuous circle. Therefore, AIDS healthcare personnel should pay attention to the self-reported health status of older adult patients to help patients improve their health and reduce the impact of the disease, to increase their level of hope.

HIV stigma and discrimination are widespread, and older PLWHA are more likely to experience ageism and sexual stigma (the sexual life of older people is considered shameful and indecent) than younger people living with HIV (5, 6).

Poor mental health due to stigma and discrimination has been well documented among people living with HIV. Thus older PLWHA with higher HIV stigma had lower level of hope. We found that older PLWHA with higher HIV stigma and lower social support have lower hope levels. Hope was also found to be positively associated with social support in other studies (24, 25). Older PLWHA have limited social networks, and in the cultural context of family responsibilities in China, family support is an important resource for older adults, who rely heavily on their families for psychosocial, financial, and caregiving support (6). Therefore, there is a need for interventions to reduce HIV discrimination in society and reduce the HIV stigma of older PLWHA. It is also important to improve family support for older PLWHA, and there is a need to monitor family members’ fulfillment of their maintenance obligations to the older adults (especially older PLWHA with low family support) and to develop family-centered measures to cope with HIV infection, to increase the level of hope for the older adults.

Our study has several strengths and limitations. To our knowledge, our study is the first study to examine the hope level of older PLWHA. But as a cross-sectional study design, this study also has several limitations. First, a definite causal relationship is difficult to obtain from a cross-sectional analysis. Second, the study was conducted in one city in China and used convenience sampling, which made the sample underrepresentative. Finally, this study ignored the possible effect of the COVID-19 epidemic on participants’ hope levels. In the future, multi-center large-scale research could be conducted in conjunction with other institutions. And longitudinal studies could be taken to track hope over time with age. Exploring the role of hope in the HIV care continuum for older PLWHA would also be meaningful.

## Conclusion

Older PLWHA had medium and high levels of hope. The AIDS healthcare health sector could use the factors influencing the level of hope of older PLWHA as an entry point to take effective measures to provide financial assistance to those in financial difficulty, help patients improve their health, reduce HIV stigma, and improve family support, thereby increasing the level of hope of patients.

## Data availability statement

The original contributions presented in the study are included in the article/Supplementary material, further inquiries can be directed to the corresponding author.

## Ethics statement

The studies involving humans were approved by the Ethics Committee of the Affiliated Hospital of Southwest Medical University. The studies were conducted in accordance with the local legislation and institutional requirements. The participants provided their written informed consent to participate in this study.

## Author contributions

CY: Writing – original draft, Methodology, Funding acquisition. YW: Writing – review & editing, Validation, Resources. YZ: Writing – review & editing, Conceptualization, Software. ML: Writing – review & editing, Methodology, Formal analysis, Software. XX: Writing – review & editing, Supervision, Project administration. LX: Writing – review & editing, Data curation, Investigation.

## Footnotes

^3^HAART: Highly active antiretroviral therapy.

## Supplementary material

The Supplementary material for this article can be found online at:

https://www.frontiersin.org/articles/10.3389/fpubh.2024.1371675/full#supplementary-material
