# Supplementary material for: Hope level and associated factors among older people living with HIV/AIDS: a cross-sectional study
Source: Front Public Health. 2024 Apr 17;12:1371675. doi: 10.3389/fpubh.2024.1371675 (PMC11061488; doi:10.3389/fpubh.2024.1371675)
Supplement: Supplementary file 1 [file Table_1.DOCX]

**Hope of older people living with HIV/AIDS**

Dear Friend:

Greetings! This survey is targeted at people living with HIV aged 50 and above, aiming to understand individual hope level. This survey is anonymous, and the relevant data will only be used for academic research, and will never be leaked, so we hope you will fill in the survey truthfully according to your own actual situation. Thank you for taking up your valuable time!

Instructions for filling in the form: Please use a ball-point pen, sign-pen or fountain pen to tick under the numbers in front of the options of each question, and fill in the corresponding contents on the horizontal line.

**General information**

1. Gender：①Male ②Female
2. Age: _________(years)
3. Education:

①Primary school and below ②Junior high school

③Senior high school ④Junior college and above

1. Household registration

①Urban ②Rural

1. Marital status

①Married ②Divorced

③widowed ④Unmarried

1. Whether is living alone

①Yes ②No

1. Personal monthly income (yuan)

①＜1000 ②1001-2000 ③2001-3000

④3001-4000 ⑤＞4000

1. Endowment insurance

①Have ②Not have

1. Length of diagnosis: ____________ (years)
2. Self-rated health

①Very poor ②Poor ③Fair

④Good ⑤Very good

1. Whether is in the stage of AIDS?

①Yes ②No

1. Have you disclosed your HIV status to family or friends?

①Yes ②No

**Chinese version Herth Hope Index (HHI)**

1. I am optimistic towards life

①completely disagree ②disagree ③agree ④completely agree

1. I have short, medium and (or) long-term goals

①completely disagree ②disagree ③agree ④completely agree

3.I feel very lonely

①completely disagree ②disagree ③agree ④completely agree

4.I can see the light amidst the difficulties

①completely disagree ②disagree ③agree ④completely agree

1. I have confidence in the treatment

①completely disagree ②disagree ③agree ④completely agree

1. I am afraid of the future

①completely disagree ②disagree ③agree ④completely agree

1. I often recall happy and pleasant times before

①completely disagree ②disagree ③agree ④completely agree

1. I feel that I have the strength to overcome the difficulties

①completely disagree ②disagree ③agree ④completely agree

1. I feel capable of giving and receiving affection/love

①completely disagree ②disagree ③agree ④completely agree

1. I feel that I should take a positive attitude and action to make things go in a good direction

①completely disagree ②disagree ③agree ④completely agree

1. I think as long as I work hard, I can play my role every day

①completely disagree ②disagree ③agree ④completely agree

1. I feel my life is very useful and worthy

①completely disagree ②disagree ③agree ④completely agree

**Social Support Scale**

1. How many close friends do you have that you can rely on for support and help? [Multiple choice] *

①0 ②1-2 ③3-5 ④6 or more

2. In the past year, you: [single choice] *

① Living away from family and living alone in one room.

② Living with strangers most of the time

③ Live with classmates, colleagues or friends.

④ Live with family

3. You and your neighbors: [multiple choice] *

① Never care about each other, just nodding friends

② May be a little concerned when faced with difficulties

③ Some neighbors: care about you

④ Most neighbors: care about you

4. You and colleagues: [Single choice] * (If no colleagues, villagers/community residents to judge)

① Never care about each other, just nodding friends

② May be a little concerned when faced with difficulties

③ Some colleagues: Very concerned about you

④ Most colleagues: very concerned about you

5. Support and care received from family members (tick "√" in the appropriate box) [table text question] *

|  | No | little | general | full support |
| --- | --- | --- | --- | --- |
| Husband and wife (lovers) |  |  |  |  |
| Parents (deceased _____ years?) |  |  |  |  |
| Sons and daughters |  |  |  |  |
| Siblings |  |  |  |  |
| Other members (e.g. sister-in-law) |  |  |  |  |

6. In the past, the sources of financial support or help in solving practical problems that you have received when you were in an emergency situation are: [Single choice]

①No source ②The following sources (multiple choices allowed):

1. Spouse; B. Other family members; C. Friends; D. Relatives; E. Colleagues;
2. Work unit; G. Official or semi-official organisations such as party groups and trade unions; H. Unofficial organisations such as religious and social groups;
3. Others —________________(please list)
4. In the past, the sources of comfort and concern you have received in times of emergency have been: [Multiple choice]

①No source ②The following sources (multiple choices allowed):

1. Spouse; B. Other family members; C. Friends; D. Relatives; E. Colleagues;
2. Work unit; G. Official or semi-official organisations such as party groups and trade unions; H. Unofficial organisations such as religious and social groups;
3. Others —________________(please list)

8. The way you talk when you encounter troubles: [single choice] *

① Never confided in anyone

② Only talk to 1-2 people who are very close to you

③ If a friend asks, you'll tell them

④ Take the initiative to talk about your troubles in order to gain support and understanding

9. How to help you when you are troubled: [Single choice] *

① Only rely on themselves, do not accept help from others

② Rarely ask for help from others

③ Sometimes ask others for help

④ Often ask for help from family, relatives and friends, and organizations when in trouble

10. Regarding the activities organized by groups (e.g., party organizations, religious organizations, trade unions, student unions, etc.), do you: [Single-choice question] *

① Never participate ② Occasionally participate

③ Frequently participate ④ Actively participate and be active

**The simplified Berger HIV Stigma Scale**

1. Some people have distanced themselves from me after they learnt that I am HIV-positive.

①agree ②disagree

2. Most of the infected people are rejected when others know that they are infected.

①Agree ②Disagree

3. Most people feel uncomfortable knowing that there are people living with HIV around them.

①agree ②disagree

4. Most people think that people with HIV are disgusting.

①agree ②disagree

5. Having AIDS makes me feel unclean.

①agree ②disagree

6. Having AIDS makes me feel like a bad person.

①agree ②disagree

7. I don't feel as good as other people (not as normal as other people) because I am infected with AIDS

①agree ②disagree

8. I try my best to keep my HIV infection a secret

①agree ②disagree

9. I never feel the need to hide the fact that I am infected with HIV

①agree ②disagree

10. Instead of telling people that I am HIV-infected, I would rather not make new friends.

①agree ②disagree

The following questions assume that you have told others about your infection status, or that others know about your infection status, which may not be the case for you, please assume that you are in this situation)

11. Once people know I'm infected, they won't want me near their children

① I agree ② I disagree

12. Once people know I am infected, they will try to avoid contact with me

① I agree ② I disagree

13. I stopped associating with certain people because of their reaction to the situation

① I agree ② I disagree

14. I'm worried that someone who knows about me will tell someone

① I agree ② I disagree

15. I was hurt by people's reaction when they found out I had AIDS

① I agree ② I disagree
